# Supplementary material for: Presence of Concurrent TP53 Mutations Is Necessary to Predict Poor Outcomes within the SMAD4 Mutated Subgroup of Metastatic Colorectal Cancer
Source: Cancers (Basel). 2022 Jul 27;14(15):3644. doi: 10.3390/cancers14153644 (PMC9332822; doi:10.3390/cancers14153644)

Supplementary Figure S1. Kaplan-Meier Curves for overall survival of metastatic colorectal cancer patients by *RAS* and *TP53* status

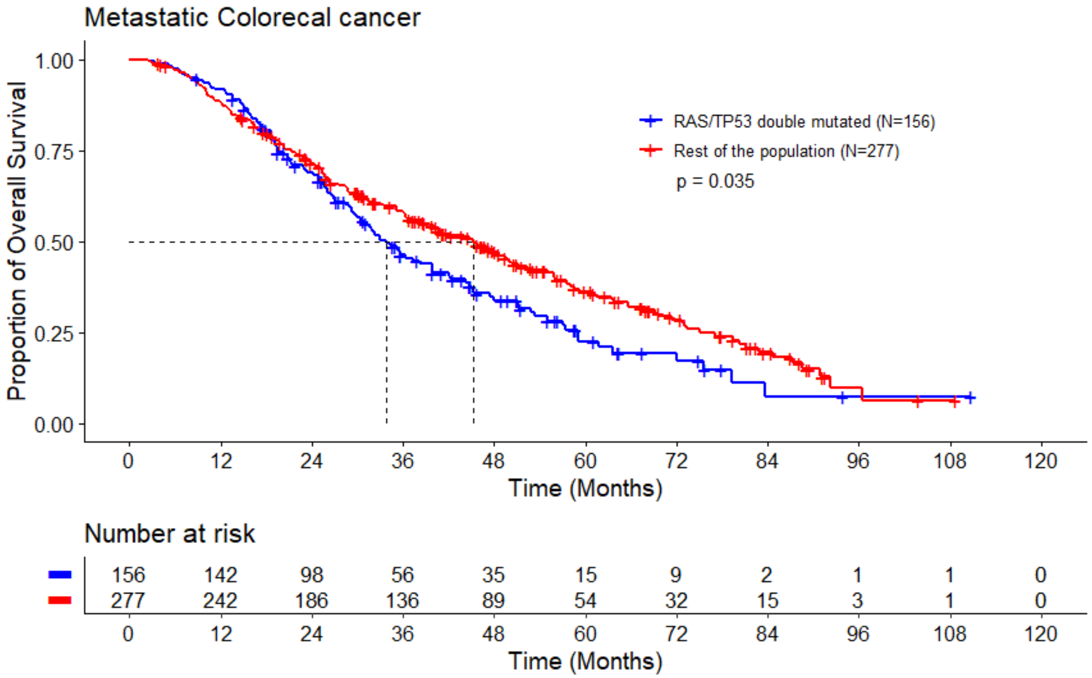

Supplementary Figure S2. Kaplan-Meier Curves for overall survival of metastatic colorectal cancer patients by *TP53* status

A

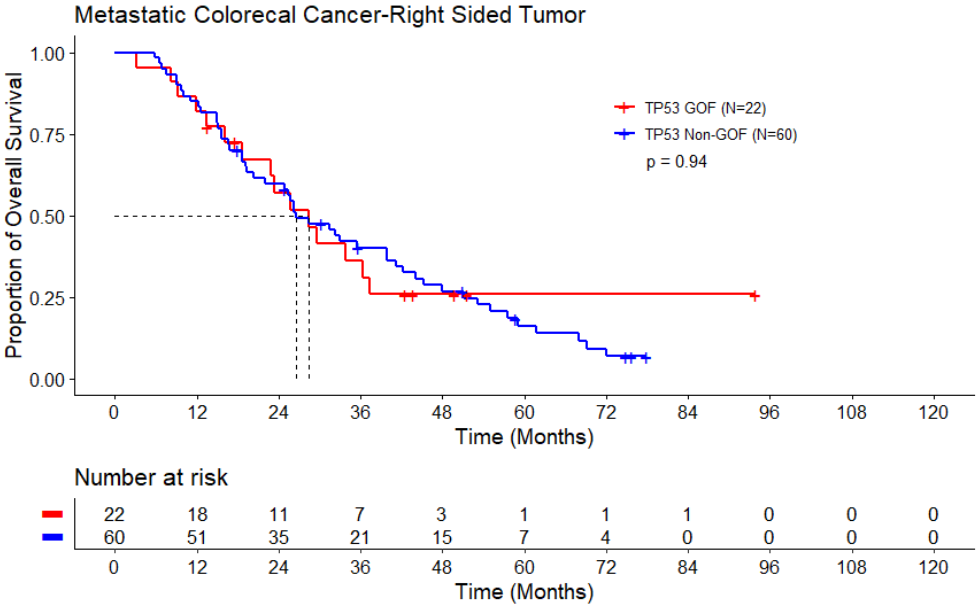

B

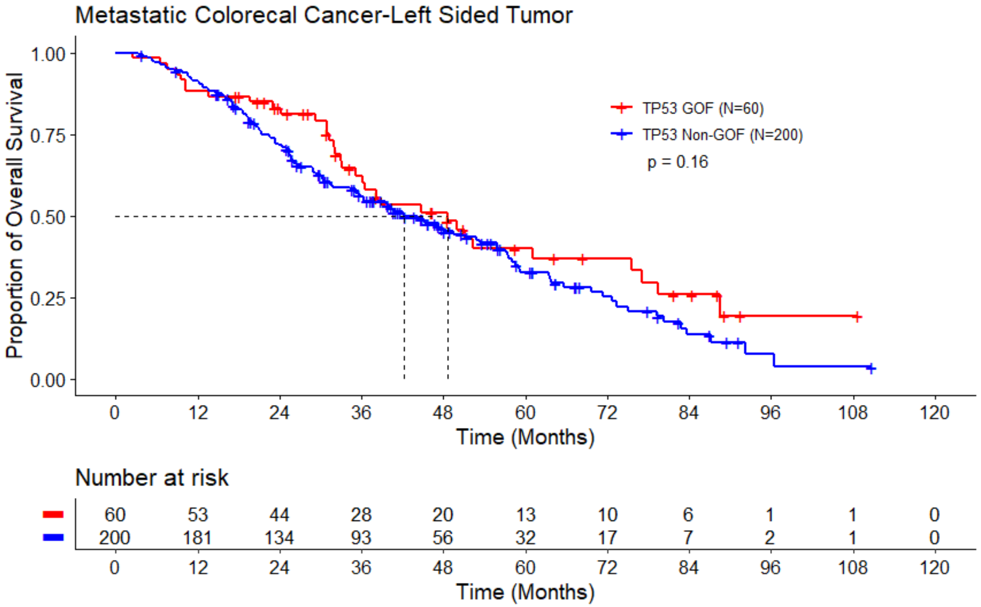

Supplementary Figure S3. Kaplan-Meier Curves for overall survival of metastatic colorectal cancer patients by *SMAD4* and *TP53* status

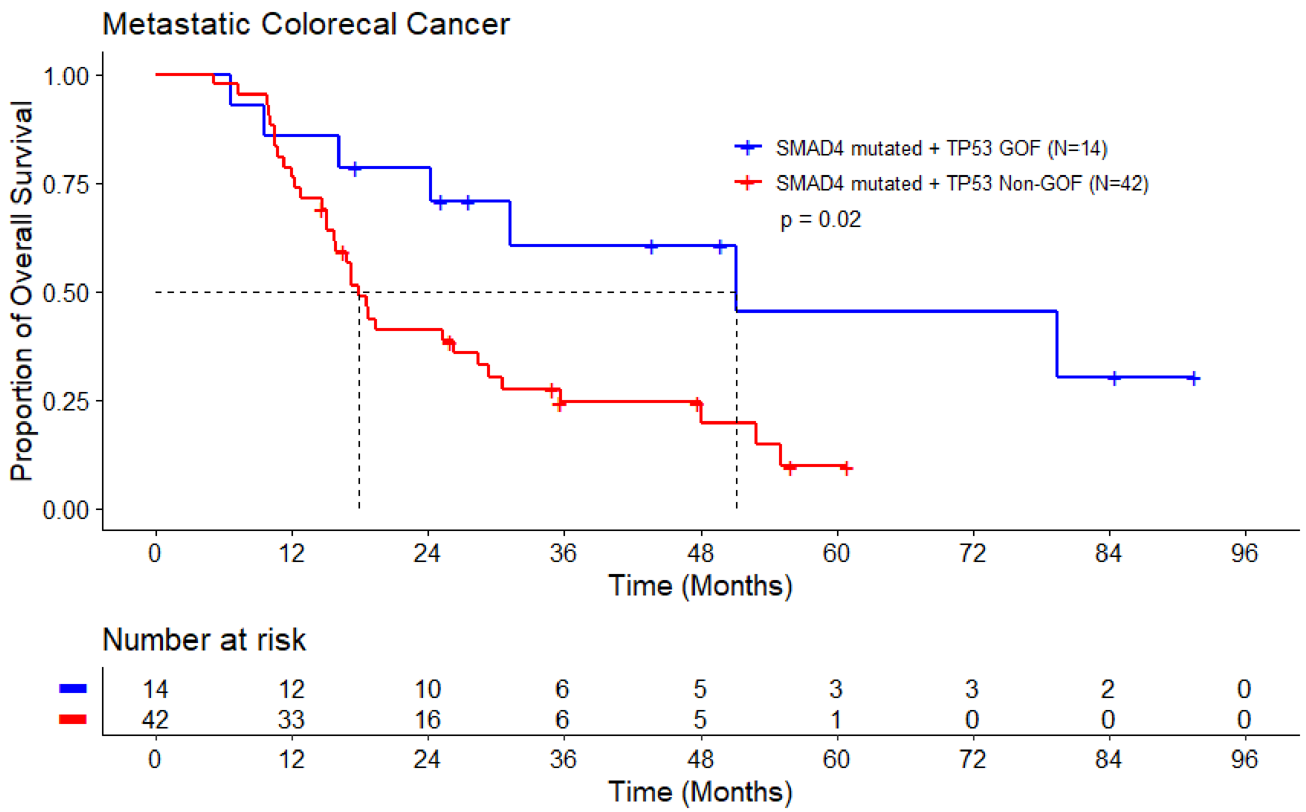

Supplement: Supplementary file 1 [file cancers-14-03644-s001.zip › Supplementary Figure.pdf]
