# Supplementary material for: Presence of Concurrent TP53 Mutations Is Necessary to Predict Poor Outcomes within the SMAD4 Mutated Subgroup of Metastatic Colorectal Cancer
Source: Cancers (Basel). 2022 Jul 27;14(15):3644. doi: 10.3390/cancers14153644 (PMC9332822; doi:10.3390/cancers14153644)
Supplement: Supplementary file 1 [file cancers-14-03644-s001.zip › Supplementary Table.pdf]

Table S1. Univariate survival model analysis for OS in 433 patients.

**Clinicopathogenic variables**	**COH cohort (n=433)**			
		**95% CI**		
	HR	Lower	Upper	P-value
Age at diagnosis (years)				
≥65 vs. <65	1.13	0.86	1.48	0.38
Gender				
Male vs. Female	1.13	0.89	1.43	0.32
Sidedness				
Right vs. Left	1.47	1.14	1.89	0.00
RAS				
Mutated vs. Non-mutated	1.15	0.91	1.46	0.24
$BRAF^{V600E}$				
Mutated vs. Non-mutated	1.72	1.13	2.62	0.01
APC				
Mutated vs. Non-mutated	0.71	0.55	0.92	0.01
TP53				
Mutated vs. Non-mutated	1.17	0.87	1.58	0.31
SMAD4				
Mutated vs. Non-mutated	1.31	0.95	1.80	0.10

Table S2. Univariate survival model analysis for OS in patients with *SMAD4* mutation.

Clinicopathogenic variables	SMAD4-MT cohort (n=70)			
		95% CI		
	HR	Lower	Upper	P-value
RAS				
Mutated vs. Non-mutated	0.98	0.54	1.76	0.94
$BRAF^{V600E}$				
Mutated vs. Non-mutated	1.55	0.65	3.68	0.32
APC				
Mutated vs. Non-mutated	0.68	0.38	1.23	0.20
TP53				
Mutated vs. Non-mutated	2.74	1.15	6.52	0.02

Table S3. Multivariate survival model analysis for OS in patients without *SMAD4* mutation.

**Clinicopathogenic variables**	**SMAD4-WT cohort (n=363)**			
		**95% CI**		
	HR	Lower	Upper	P-value
RAS				
Mutated vs. Non-mutated	1.26	0.96	1.66	0.10
$BRAF^{V600E}$				
Mutated vs. Non-mutated	1.64	0.95	2.84	0.08
APC				
Mutated vs. Non-mutated	0.80	0.58	1.10	0.17
TP53				
Mutated vs. Non-mutated	1.10	0.79	1.53	0.57
